# A Note on Enhancing Aeration via a Vortex-Based Cavitation Device

**DOI:** 10.1021/acsomega.4c08452

**Published:** 2025-02-02

**Authors:** Jagdeep Kumar Nayak, Amol Ganjare, Vivek V. Ranade

**Affiliations:** Multiphase Reactors and Intensification Group Bernal Institute, University of Limerick, Limerick V94 T9PX, Ireland

## Abstract

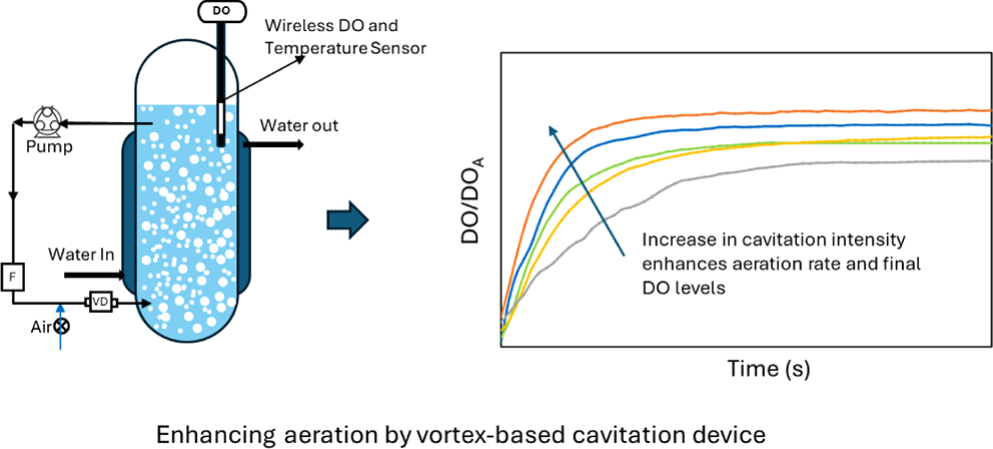

There is growing interest in generating micro- or nanobubbles for enhancing aeration. Small bubbles not only enhance the interfacial area for gas–liquid mass transfer but also may enhance the equilibrium solubility if the size of the bubbles is small enough. In this note, we demonstrate the use of a vortex-based hydrodynamic cavitation device (VD) for generating small bubbles and enhancing aeration. Experimental results for conventional aeration and aeration with VD operated under three different conditions are presented. A reference case of potential degassing because of the low pressure generated in the cavitation device was also investigated. Experiments were carried out in a bubble column using DI water as the liquid phase. The dissolved oxygen (DO) concentration was measured using a precalibrated dissolved oxygen probe. Measurements of transient profiles of dissolved gas concentrations were carried out under different operating conditions. A generalized framework to analyze mass transfer in the presence of degassing, absorption, and desorption (via top surface or large bubbles) is developed and used for interpreting the experimental data. The per-pass degassing factor of VD was found to increase with the power dissipation [∝ (*P*–*P*_c_)^0.4^, where *P* is power dissipation and *P*_c_ is the critical power beyond which degassing starts]. The aeration generated by VD was found to realize 30% higher DO concentration beyond the equilibrium solubility at atmospheric conditions. The bubble sizes estimated from the steady-state DO concentration were in the range from 80 to 200 μm for the operating parameters considered in this work. The presented results demonstrate the effectiveness of VD for enhancing aeration and will be useful for intensifying gas–liquid processes.

## 1 Introduction

Aeration by sparging gas into a pool of liquid is used in a variety of applications where oxygen mass transfer from sparged air to the liquid phase is crucial. Some examples where aeration is used include water treatment,^1^ food processing,^2^ biorefinery,^3^ aerobic fermentation,^4^ etc. The effectiveness of the oxygen transfer is often evaluated based on the steady-state dissolved oxygen (DO) concentration and the time required to achieve it. The aeration performance thus depends on the mass transfer coefficient, interfacial area, and concentration driving force. Smaller bubbles lead to a lower mass transfer coefficient and larger interfacial area. In most cases, the enhancement in the effective interfacial area (m^2^/m^3^) with smaller bubbles is far larger than the reduction in the mass transfer coefficient. Therefore, the effective aeration rate increases with the reduction in bubble sizes. Most of the attempts for enhancing aeration therefore focus on increasing the interfacial area, that is, reduction in bubble sizes.

Several spargers and ways of introducing air into the pool of liquid have been proposed (see, for example, Wang et al.^5^ and references cited therein). Recently, Desai and Zimmerman^6^ have reviewed state-of-the-art methods for generating microbubbles. This review provides a broad overview of the current state of the art on microbubble generation. We would like to highlight a couple of studies here that are relevant to the case considered in the present work. Yao et al.^7^ used a conventional bubble generator to study the effects of micronano aeration on nitrification. During the aeration process, they measured *k*_L_*a* and the maximum DO levels, which were found to be 0.56 min^–1^ and 11.9 mg/L, respectively. Lu et al.^8^ conducted a study to examine the impact of nanobubble aeration on DO levels and nitrogen removal. They observed a maximum DO concentration of 10.14 mg/L, reached within 20 min, with a *k*_L_*a* value of 0.3 min^–1^. Levitsky et al.^9^ demonstrated that under oscillating flow conditions, microbubbles increase the volumetric mass transfer coefficient (*k*_L_*a*) by about 45%, achieving faster oxygen saturation than conventional bubbles. Similarly, Pambudiarto et al.^10^ found that optimizing flow rates in an orifice/porous-pipe microbubble generator maximizes oxygen dissolution, with higher liquid flow rates leading to more uniform bubble sizes and a *k*_L_*a* plateau of 0.07 s^–1^. Nyoman Suwartha et al.^11^ confirmed that smaller microbubble sizes, such as 89 μm, result in higher *k*_L_*a* values and extended aeration contact time, both beneficial for efficient mass transfer. Additionally, microbubbles significantly improve the COD degradation rates in wastewater treatment. Studies have shown that microbubble generators can increase COD removal by 2–5.9 times compared to conventional bubbles, making them highly effective for enhanced wastewater treatment processes.^12^

Hydrodynamic cavitation offers an excellent way to generate smaller droplets or bubbles.^13,14^ In this work, we demonstrate the effectiveness of a vortex-based hydrodynamic cavitation device (VD) for the generation of microbubbles to enhance aeration performance. A loop configuration is used for carrying out aeration experiments in a bubble column operated in a semibatch mode, similar to that used for generating oil-in-water emulsions by Gode et al.^15^ The transient profiles of the DO concentration and steady-state DO concentration were measured at different operating conditions. The developed generalized framework to analyze mass transfer in the presence of degassing, absorption, and desorption (via the top surface or large bubbles) will be useful for interpreting various techniques for enhancing aeration performance. The presented approach and results will be useful to effectively harness hydrodynamic cavitation for intensifying gas–liquid reactors and processes.

## 2 Experimental Setup, Procedure, and Data Processing

### 2.1 Setup, Materials, and Experimental Procedure

**Figure 1:**
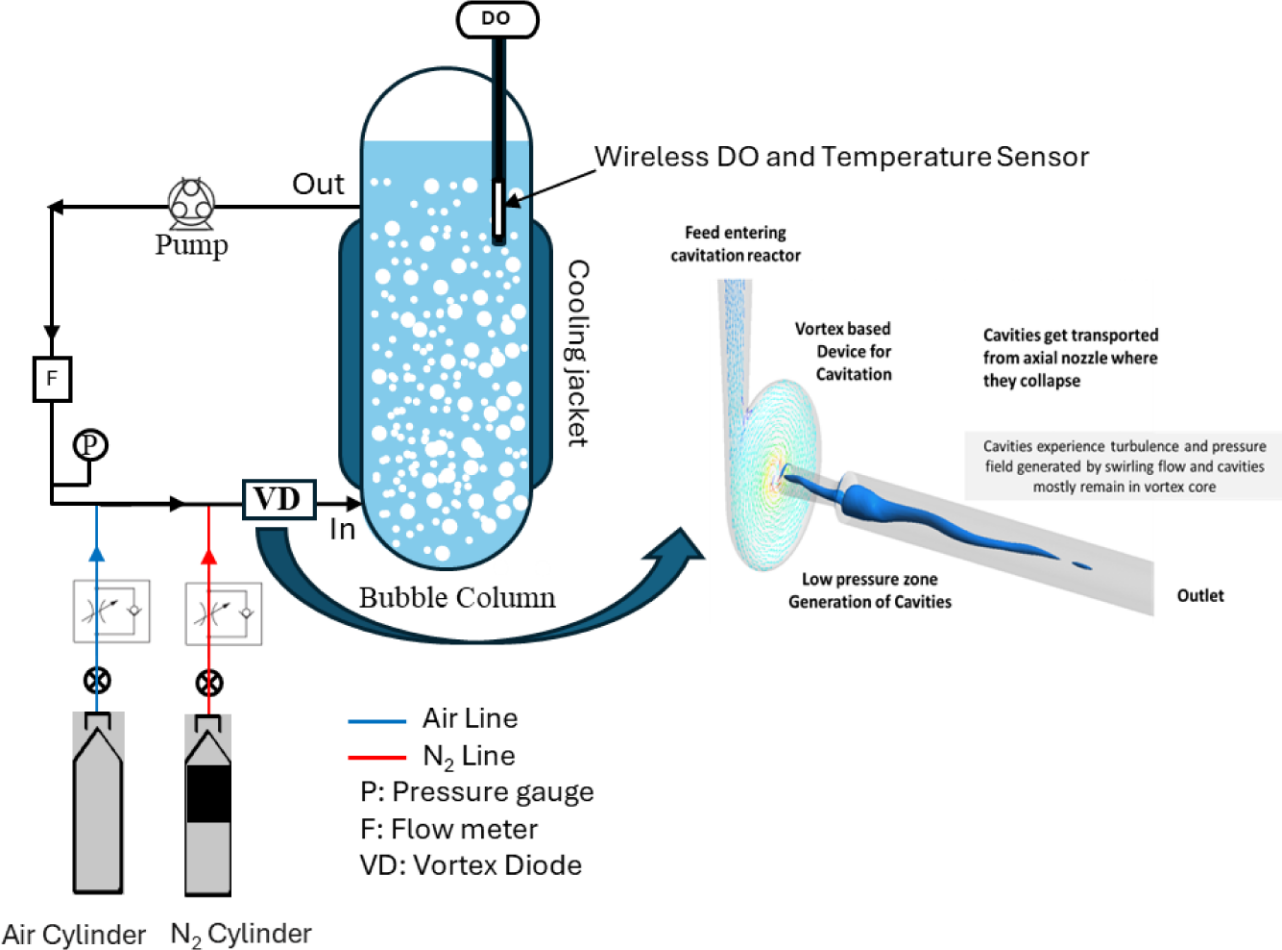
Schematic of the experimental setup.

The experiments for conventional aeration, desorption, and small bubbles enhancing aeration with the VD were performed in the bubble column setup. The arrangement of the bubble column, the loop configuration, and placement of the sensor are shown schematically in Figure 1. The vortex-based cavitation device,^14,15^ henceforth abbreviated as VD, was used for generating microbubbles. The geometrical details of VD^15^ and the bubble columns are provided in the Supporting Information (see Figure S1a,b). The photograph of the setup is shown in Figure S2a.

**Figure 2:**
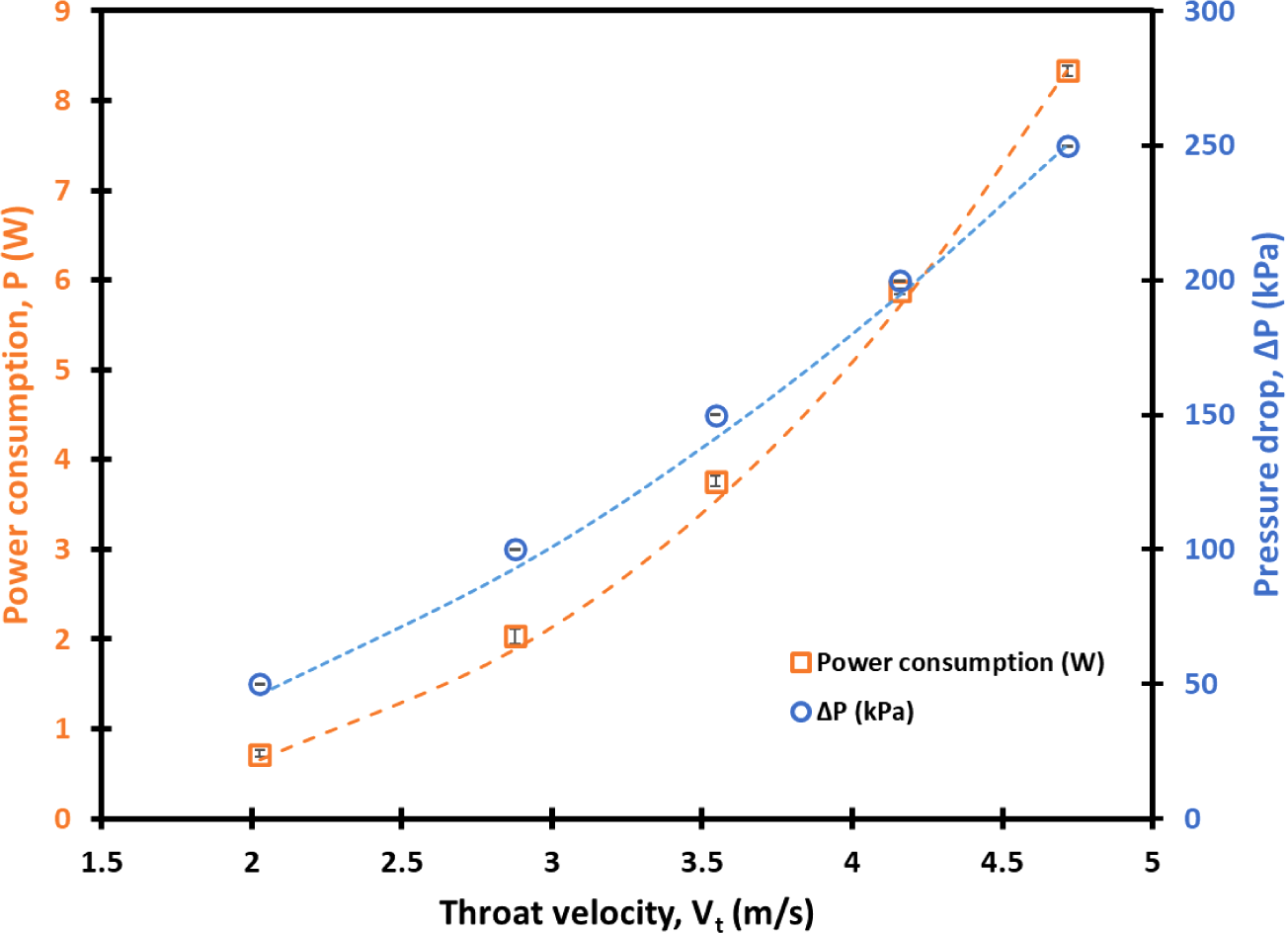
Pressure drop and power consumption characteristics of the VD. Symbols denote experimental data, and dashed lines denote correlations. Blue: eq 11 and orange: eq 12.

The DO was measured with a wireless optical dissolved oxygen sensor (PS-3246) purchased from Pasco Ireland. The maximum range of this sensor was 20 mg/L with an accuracy of ±0.2 mg/L. This sensor was able to compensate for the temperature automatically, and the data were logged wirelessly on a separate device. The values of the DO concentration, percent oxygen saturation, and temperature were collected for all the experiments. Deionized water produced with the Barnstead Smart2Pure Water Purification System provided by Thermo Scientific was used in all the experiments (physical properties of water and air are mentioned in Table S2). The bubble column was maintained at 20 °C by circulating water through the jacket of the bubble column. The cavitation device, VD, was initially characterized by measuring the pressure drop for different flow rates. The flow rate and pressure drop were monitored throughout the experiments with a digital flow meter (Krohne AF-E 400) and Bourdon tube pressure gauge (Wakai Model 111, 0–10 bar), respectively. The measured pressure drops as a function of throat velocity, *V*_t_ (flow rate divided by the throat area of the VD^14^) are shown in Figure 2.

The pressure drop results were correlated using eq 1, and the value of the Euler number (Eu) was found to be 23. The pressure drop values in the presence of gas were measured experimentally, and the data indicate that gas flow increases the Euler number by about 15% (Eu = 27; see Figure S3 and Table S5).
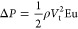
1where *ΔP* is the pressure drop across the vortex-based cavitation device, *V*_t_ is the throat velocity, and ρ is the density of the fluid. The power consumption, *P*, can then be calculated as
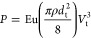
2

where *d*_t_ denotes the throat diameter of the vortex-based cavitation device.

After characterization of the VD, three sets of experiments were carried out. The first set of experiments focused on characterizing the degassing performance of the VD. It is well-known that the VD generates a strongly swirling flow in the vortex chamber and creates a low pressure region where cavitation initiates.^14,15^ This low-pressure region also causes degassing (desorption of dissolved gases from water). It is therefore essential to quantify the degassing effect of the VD and appropriately account for it while interpreting aeration results. These experiments were carried out by first saturating the water in the bubble column with air by sparging air. A silicon carbide sparger of size 15 × 25 mm was used (see Figure S2b). Once the water is saturated with air, the DO level stabilizes. The air flow is then stopped, and the flow through the VD loop is started. As the saturated water starts to flow through the VD, it gets exposed to the low-pressure region, and the DO level in the bubble column starts falling. The reduction in the DO level with time was measured for three pressure drops across the VD in the range of 150 to 250 kPa (flow rate ranging from 2.5–3.3 × 10^–5^ m^3^/s). These results are reported and discussed in Section 3. As the pressure drop across the cavitation device increases, the number density of cavities generated in the device increases. However, the intensity of cavity collapse decreases with an increase in the pressure drop.^14,16^ Because of these two competing effects, it is expected that beyond a certain pressure drop, the performance will plateau or even possibly decrease. Previous studies on water treatment, droplet breakage, and pretreatment of biomass slurries have indicated 250 to 300 kPa as an optimum pressure drop. However, a previous study identified the inception of cavitation between a 50 and 100 kPa pressure drop across the HC device.^17^ The value of the optimum pressure drop is expected to change with specific processes under consideration. However, based on prior studies and constraints of the experimental setup used in the present work, we restricted the maximum pressure drop across the device to 250 kPa.

Aeration experiments were then carried out using the conventional way as a reference case (air introduced through a sparger) and using the VD, where gas is introduced at a flow rate of 0.2 LPM in the flow loop before the VD, as shown in Figure 1. Before the aeration experiments were started, the DO concentration was reduced by purging nitrogen through the bubble column via a sparger. Nitrogen was used considering its ready availability in the laboratory. The rate of reduction of DO was found to be significantly low as the DO approached 0.3 ppm. The DO concentration is not expected to attain zero since the top surface of the container was exposed to air. Considering these factors, it was decided to stop the stripping operation as the DO approached 0.3 ppm. Once the minimum DO level remained steady for a significant time, the nitrogen supply was shut off and air was supplied to the bubble column (either through a sparge or through the VD). The DO level started rising. The transient profiles of DO were measured for different operating conditions of the VD. All of the experiments were repeated at least three times and were found to be reproducible. Average values of the DO were used for further processing and interpretation of results. The framework for interpreting the results is discussed in the following section.

### 2.2 Processing of Experimental Data

The measured DO profiles indicate that if the experiments are conducted long enough, the DO level attains a stable value at the steady state. This was observed for both the degassing as well as aeration experiments (for conventional as well as the VD). Assuming that Henry’s law is applicable for describing DO concentration in water, the equilibrium DO solubility () may be assumed to be linearly proportional to prevailing partial pressure (, where *H*’ is Henry’s constant for the oxygen–DI water system, *P*_A_ is the atmospheric pressure, and *y*_O_ is the mole fraction of oxygen). In the present case, the oxygen mass fraction was assumed to be constant in all the experiments, and therefore, gas phase mass balance was not considered. The equilibrium solubility of oxygen at atmospheric pressure () can therefore be simply written as , where *H* = 0.21*H*’. The steady DO level at the steady state during the degassing experiments was found to be much higher (8.27 mg/L, more than 85% of atmospheric solubility) than the equilibrium solubility at low degassing pressures (∼10% of atmospheric solubility). The absolute values of DO (mg/L) are stated (Table S1). This indicates that there has to be an absorption of oxygen to compensate for degassing occurring in the VD. The top surface exposed to the atmosphere and some of the air bubbles escaping low-pressure zones will cause reabsorption of oxygen into the degassed liquid. The balance of these two processes, degassing and reabsorption (either from the top surface or bubble escaping the low-pressure zone), causes a steady value of DO concentration, which is much higher than the equilibrium solubility at the low degassing pressures.

Similarly, when air is introduced through the VD, hydrodynamic cavitation is expected to generate microbubbles only if it is operated beyond the cavitating regime. The pressure inside the bubble (*P*_B_) is given by the following equation:^18^
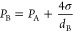
3

Where *P*_A_ is the surrounding pressure (considered as atmospheric pressure in this work, since the static liquid head is negligible compared to the atmospheric pressure), σ is the surface tension (0.072 N/m for the air–water case)^23^ and *d*_B_ is the bubble diameter. Considering the constant mole fraction of oxygen, the concentration of DO is directly proportional to the pressure. Therefore, by dividing the DO concentration in the bubble () by the expected DO concentration in a bubble at atmospheric pressure  gives
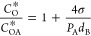
4

The values of this concentration ratio for bubbles of 1, 10, and 100-μm diameter are 3.85, 1.29, and 1.03. The earlier results of oil-in-water emulsion^15,19^ indicate that cavitation generates drops of around 1 μm. If air bubbles of 1 mm are generated, the equilibrium saturation level may go as high as 3.85 times that of atmospheric saturation. However, the absorption (from the top surface or from the bubble escaping the low-pressure zone) observed in the case of degassing experiments will cause desorption in this case and will lower the prevailing DO concentration. Considering this physical picture, liquid phase balance equations are written by considering three steps:

- Degassing by the VD: influence of the low-pressure zone and resulting degassing was modeled using the per-pass approach.^14^ The DO level was assumed to be reduced by a factor (1–⌀) in every pass through the VD.
- Mass transfer from bubbles to the liquid: this is modeled in a conventional way by introducing the volumetric mass transfer coefficient (*k*_L_a) and concentration driving force. Depending on the bubble diameter, the equilibrium DO concentration was calculated using eq 4.
- Mass transfer from the liquid to bubbles (top surface or bubbles escaping low pressure regions): this is modeled in a similar way using a separate value of the volumetric mass transfer coefficient (*k*_L_*a*’). The saturation concentration is assumed to be internal pressure is assumed to be .

The dimensionless mass balance of oxygen in the liquid phase can thus be written as
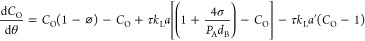
5

Where, *C*_O_ is a dimensionless liquid phase DO concentration (normalized by ), θ is dimensionless time () and ⌀ is the per-pass degassing coefficient. *V* is the volume of the bubble column, including the tubing, *Q* is the volumetric flow rate through VD, and *t* is time. This can be solved as
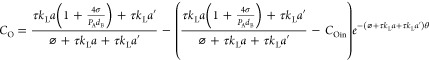
6

Where *C*_Oin_ is the initial dimensionless DO concentration (θ = 0).

This generalized equation can then be used to interpret degassing as well as aeration experiments as special cases. For example, in the degassing experiments, the equation is reduced to eq 7 by setting τ*k*_L_*a* to zero (by considering *C*_Oin_ = 1):
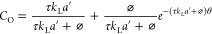
7

The steady state DO concentration (*C*_Os_) can thus be calculated as
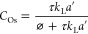
8

The experimentally measured value of steady state DO concentration (*C*_Os_) can be used to relate ⌀ and τ*k*_L_*a*’. The transient profiles of the DO concentration can then be described by fitting one of these parameters. These results are discussed in Section 3.

For the case of conventional aeration and aeration through the VD, the steady-state DO concentration may be written as
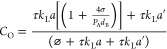
9

The values of parameters ⌀ and τ*k*_L_*a*’ obtained from degassing experiments can be used. Thus, the only other two unknown parameters, τ*k*_L_*a* and *d*_B_, are related by eq 9. The transient profiles of DO concentration can then be described by fitting one of these parameters. These results are discussed in Section 3.

## 3 Results and Discussion

The aeration is enhanced by the small bubbles generated by hydrodynamic cavitation in the bubble column. Hydrodynamic cavitation also leads to degassing. The impact of degassing and the aeration through the VD on the dissolved oxygen in water is discussed in this section. The correlations of the degassing coefficient and mass transfer coefficients for cavitating devices in terms of power consumption and critical power consumption are presented.

### 3.1 Degassing by the VD

**Figure 3:**
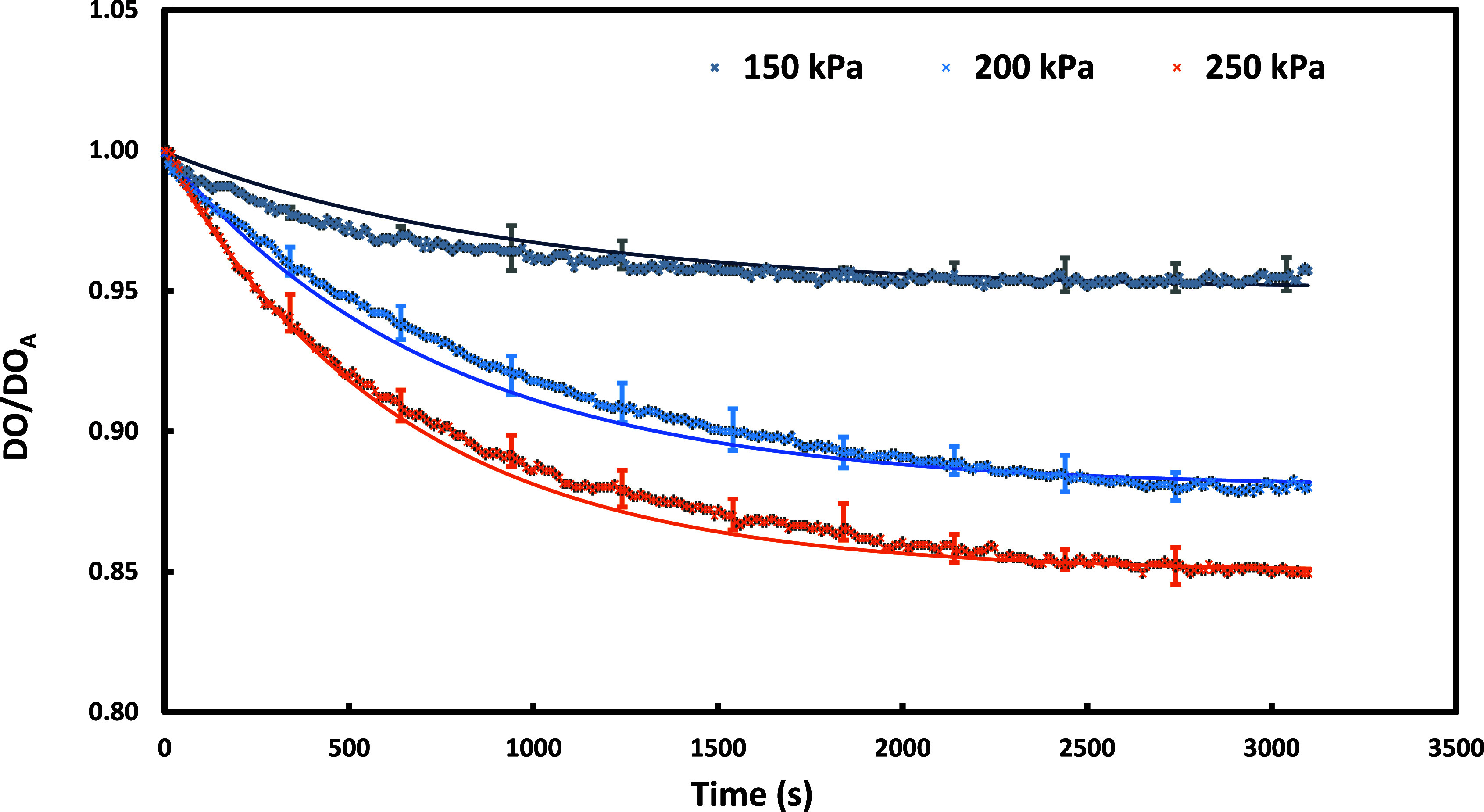
Transient profiles of normalized DO concentration for degassing experiments with the VD. Symbols denote experimental data. Continuous lines indicate fitted profiles (eq 7). *To avoid clutter, the error bars are provided at intermediate points.

The effects of hydrodynamic cavitation leading to degassing can be quantified through the depletion of normalized DO. The measured transient profiles of normalized DO concentration under degassing conditions (no air–only flow through the VD) are shown in Figure 3 for the different pressure drops across the VD.

**Figure 4:**
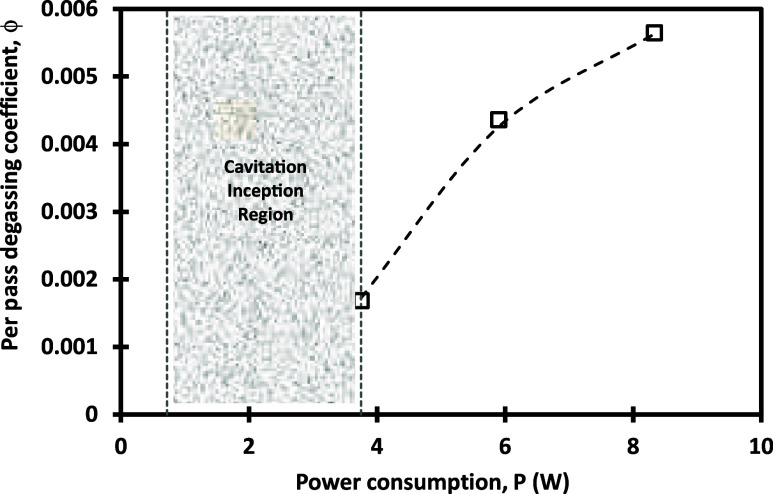
Per-pass degassing coefficient for the VD. Symbols denote fitted values, and dashed line indicates eq 10.

It can be seen that a higher pressure drop across (or flow rate through) the VD leads to higher degassing. At a pressure drop of 250 kPa, the steady-state DO concentration was found to be 15% lower (8.29 mg/L) than the usual saturation concentration. The steady-state DO concentration and transient profiles measured in these experiments were used to obtain parameters appearing in eq 7. The parameter τ*k*_L_*a*’ was found to be 0.032 for all the cases. This agrees with the intuitive understanding that this parameter is not expected to be a function of flow through the VD. The fitted value of the per-pass degassing coefficient was found to increase with the pressure drop across VD. The fitted values of the per-pass degassing coefficient are shown as a function of power consumption in Figure 4.

The behavior of the per-pass degassing factor with power consumption may be approximated as
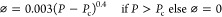
10

Where *P*_c_ is the critical power consumption (W) beyond which degassing starts. The objective of the proposed fitting was to discuss the observed trends rather than to provide an empirical equation for design. Two key trends were highlighted: (a) existence of critical power consumption (*P*_*c*_) beyond which significant degassing starts, and (b) the degassing coefficient was found to be proportional to (*P*–*P*_c_)^0.4^ where *P* is the power consumption. When the VD is operated with power consumption less than *P*_c_, the per-pass degassing coefficient is very low. These fitted values, per-pass degassing factors, and τ*k*_L_*a*’ were used for interpreting aeration results using the VD. As shown in Figure 4, the per-pass degassing coefficient increases with an increase in the pressure drop across the vortex-based cavitation device. The dependence of the per-pass degassing coefficient is related to power consumption (which is a product of pressure drop and flow rate) as eq 10 which captures the combined influence of higher degassing because of a larger liquid volume per pass and higher degassing because of a higher extent of cavitation.

### 3.2 Aeration with and without the VD

**Figure 5:**
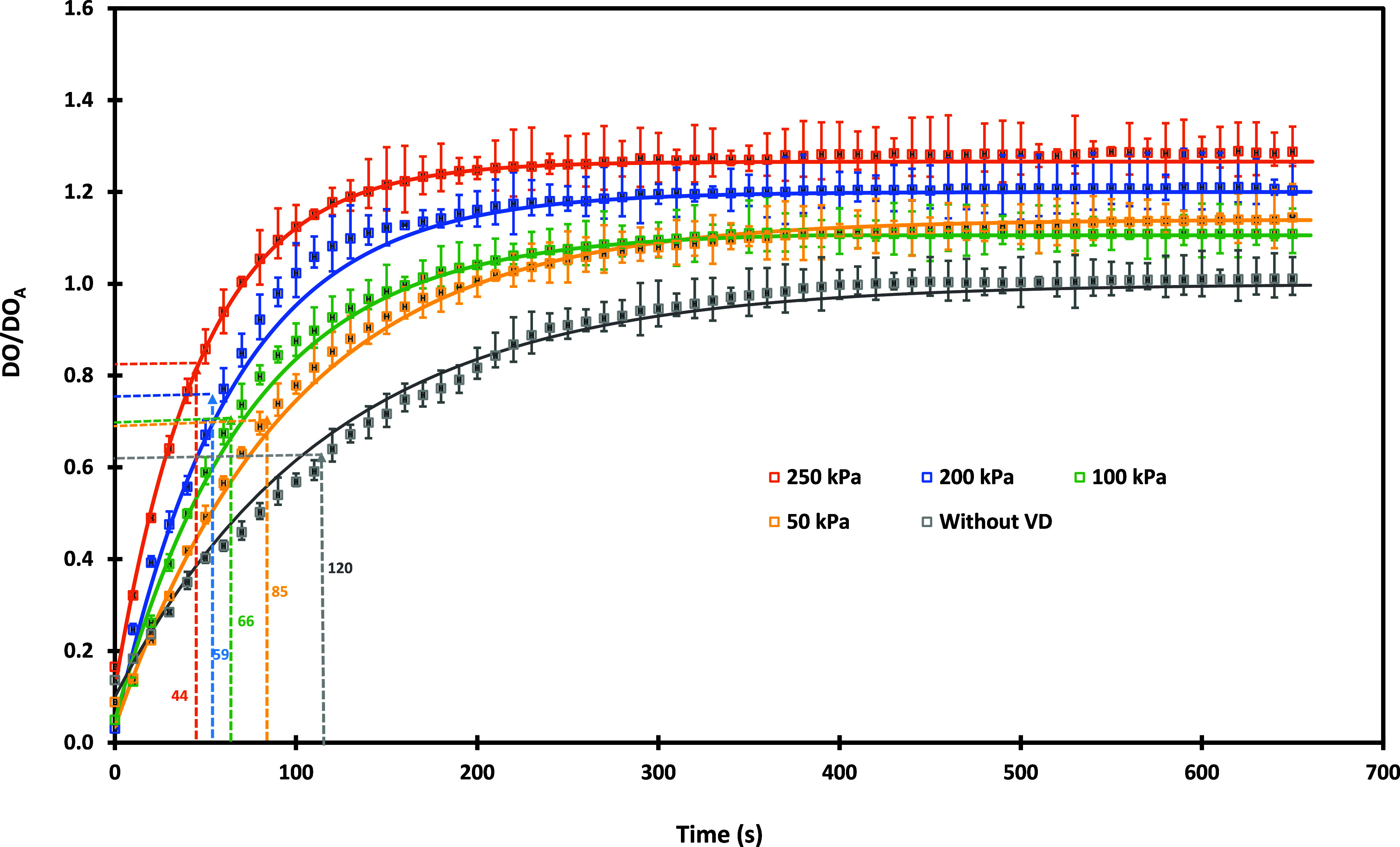
Transient profiles of DO concentration for aeration with and without the VD. Symbols denote experimental data, and continuous lines indicate fitted profiles (eq 9).

As discussed earlier, the water in the column was initially stripped of oxygen with nitrogen, and after reaching a stable DO concentration (0.3 ppm), air was passed to the bubble column. The experimentally measured transient profiles of DO concentration (normalized by DO solubility under atmospheric conditions, DO_A_) without and with the VD at different pressure drops are shown in Figure 5. It can be seen from Figure 5 that when conventional aeration was conducted (air sparging through a sparger), the steady-state DO (normalized) approaches one. The rise in DO concentration is rather slow (compared to the results obtained with the VD), and the time required to reach 63% of the maximum DO concentration was found to be 120 s for aeration without the VD. When air was introduced through VD, the rise in DO concentration was faster than without the VD. The steady-state DO concentration was higher than one and was found to increase with an increase in pressure drop across the VD. The time required to reach 63% of the maximum DO concentration was also found to decrease with pressure drop. These values were found to be 85, 66, 59, and 44 s for pressure drops of 50, 100, 200, and 250 kPa, respectively. As the pressure drop across the cavitation device increases, the number density of cavities generated in the device increases. However, the intensity of cavity collapse decreases with an increase in pressure drop.^15^ Because of these two competing effects, it is expected that beyond a certain pressure drop, the performance will plateau or even possibly decrease. Previous studies on water treatment, droplet breakage, and pretreatment of biomass slurries have indicated that 250 to 300 kPa is an optimum pressure. The value of optimum pressure is expected to change with the specific process under consideration. However, based on prior studies and constraints of the experimental setup used in the present work, we restricted the maximum pressure drop across the device to 250 kPa.

**Figure 6:**
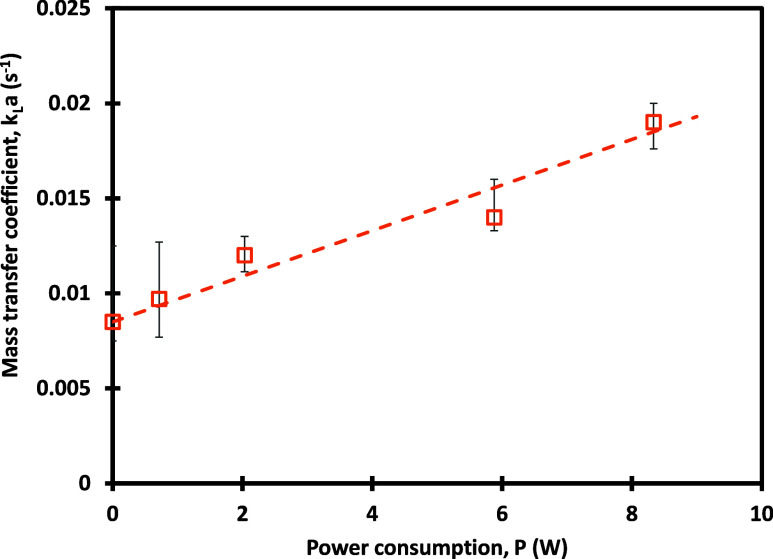
Fitted values of the mass transfer coefficient without and with the VD. Symbols denoted fitted values, and the dashed line indicates eqs 11 and 12.

This shows that the VD reduced the time required to reach the higher dissolved oxygen concentration along with achieving higher dissolved concentrations as compared to conventional aeration. The fitted values of *k*_L_*a* for experiments without and with the VD are shown in Figure 6. It should be noted that vortex-based cavitation leads to a significant enhancement in mass transfer. For example, the *k*_L_*a* reported by van de Griend et al. (2022)^20^ and Park et al. (2022)^21^ are less than 11 h^–1^ unlike the present work where values up to 70 h^–1^ have been observed. Table 1 presents a comparison of *k*_L_*a* values to those of other vortex-based microbubble generators.

**Table 1:** Comparison of *k*_L_*a* with Published Studies

Microbubble generator	Maximum reported *k*_L_*a* (h^–1^)	Pressure drop (kPa)	Reference
vortex impeller-based aeration	10.5	complete regime (see the cited reference for more details)	(20)
vortex aerator	2.7	150	(21)
multistage vortex aerator	25	150	
venturi bubble generator	20	400	(22)
vortex sparger	11	60	(11)
upper venturi	10	170	
lower venturi	11	240	
vortex-based hydrodynamic cavitation	70	250	this study

It can be seen that the trend observed for the fitted values of the volumetric mass transfer coefficient may be approximated by the following linear equation:
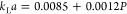
11

The characteristic time scale of the rise in the DO may be approximated as an inverse of *k*_L_*a*. The fitted values of the effective bubble diameter obtained from the transient profiles of DO concentrations may be represented as
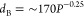
12

This indicates that the effective bubble diameter is 170 mm when power consumption is 1 W and is proportional to *P*^–0.25^. The observed proportionality is similar to that reported for oil-in-water case by Gode et al.^15^ An attempt was also made to measure the bubble size distribution using an endoscopic probe (SoPAT–PL imaging probe) with the addition of a surfactant. The presence of surfactant, however, reduced interfacial tension, and the bubble sizes measured in the presence of surfactants were found to be less than 100 μm at a 200 kPa pressure drop (see Figure S2c,d). The measured bubble sizes are less than 170 μm because of the presence of surfactant as well as higher power consumption at a 200 kPa pressure drop. The vortex-based cavitation device, VD, was able to generate fine bubbles and enhance aeration performance. As mentioned earlier, the DO increases because of the combined effect of pressure drop (more intense cavitation–smaller bubbles–higher inside pressure–higher equilibrium concentration) and flow rate (larger liquid volume per pass). The results indicate the superiority of aeration using the VD in terms of maximum DO concentration achievable (1.3 times equilibrium solubility under atmospheric pressure) and the time required to achieve it.

It will be useful to include here some comments on scale-up. Typically, bubble columns exhibit the relationship between volumetric mass transfer coefficient (*k*_L_*a*) and gas superficial velocity (*V*_G_) as  where β varies in a narrow range of 0.85 to 1.15 (Deshpande et al.^24^ and references cited therein). Considering this, maintaining the same superficial velocities across the scales is generally considered a reasonable scale-up strategy. Please note that several other factors, such as column aspect ratio, sparger configuration, and prevailing flow regimes, may influence scale-up in a complex way. Further discussion on the scale-up of bubble columns is not included here since the focus of the present note is on enhancing aeration effectiveness by generating microbubbles. In this work, we have demonstrated the effectiveness of a vortex-based hydrodynamic cavitation device for the generation of microbubbles to enhance aeration performance. The discussion on scale-up is, therefore, limited to the scale-up of the cavitation device (to maintain a constant Sauter mean diameter of bubbles across different scales of the cavitation device) rather than the scale-up of the bubble column reactor (to maintain constant *k*_L_*a* across different scales of the bubble column reactor). The effectiveness of the device depends on the generation of microbubbles via cavitation. If the cavitation device is scaled up to ensure the generation of adequately small bubbles, then effectiveness is not expected to depend on the size of the bubble column. It has been shown that the influence of device scale (over the range of nominal flow rate from 1.2 LPM to 20 LPM) maintains effectiveness for droplet breakage.^15^ Similar behavior is expected for the breakage of the gas bubbles. In this work, we have used a gas flow rate of approximately 10% of the liquid flow rate. Therefore, the larger-sized device with a similar ratio of gas to liquid flow rates will generate similarly sized bubbles and therefore lead to similar mass transfer performance per unit energy consumption. We hope that the approach and results presented here will stimulate further work in this promising research direction.

## 4 Conclusions

In this work, a vortex-based hydrodynamic cavitation device, VD, was used for generating small bubbles and enhancing aeration. The dissolved oxygen concentrations were measured for degassing in the VD, aeration with the VD, and aeration with and without the VD. A generalized framework for analyzing the degassing and aeration in the VD was developed, and dissolved oxygen profiles were predicted. The VD was found to accelerate the dissolution and enhance the dissolved oxygen content beyond equilibrium solubility under atmospheric conditions. The key conclusions are listed below.

- The VD was found to degas and reduce DO concentration. The extent of reduction increases with a pressure drop across the VD.
- The per-pass degradation factor was found to be proportional to excess (to critical power consumption) power consumption raised to 0.4 (Equation 9).
- The VD intensified the aeration and led to a faster rise in DO concentration compared to aeration without the VD. The extent of intensification increases with increase in a pressure drop across the VD.
- The effective mass transfer rate was found to exhibit two regimes similar to the regimes observed in the case of the per-pass degassing factor. Equation 10 may be used to estimate the effective mass transfer coefficient.
- The effective bubble diameter was found to be proportional to *P*^–0.25^ with a proportionality constant of 170 μm for a power consumption of 1 W.

The approach and generalized framework developed here will be useful for harnessing hydrodynamic cavitation, particularly vortex-based cavitation devices, for intensifying aeration.

## Supporting Information Available

Details of the vortex-based cavitation device and bubble column, photograph of experimental setup, image of microbubbles, bubble size distribution, pressure drop characteristics of the cavitation device, initial and final DO values for degassing experiments, physical properties, operating conditions, and the cavitation number at different pressure drops; derivation of eq 3 (PDF)
