# Supplementary material for: A Note on Enhancing Aeration via a Vortex-Based Cavitation Device
Source: ACS Omega. 2025 Feb 2;10(5):4561–8. doi: 10.1021/acsomega.4c08452 (PMC11822708; doi:10.1021/acsomega.4c08452)
Supplement: Supplementary file 1 — ao4c08452_si_001.pdf [file ao4c08452_si_001.pdf]

Supplementary information

A Note on Enhancing Aeration via Vortex-based Cavitation Device

Jagdeep Kumar Nayak, Amol Ganjare and Vivek V. Ranade*
Multiphase Reactors and Intensification Group
Bernal Institute, University of Limerick, Ireland
*Email: Vivek.Ranade@ul.ie

(a)

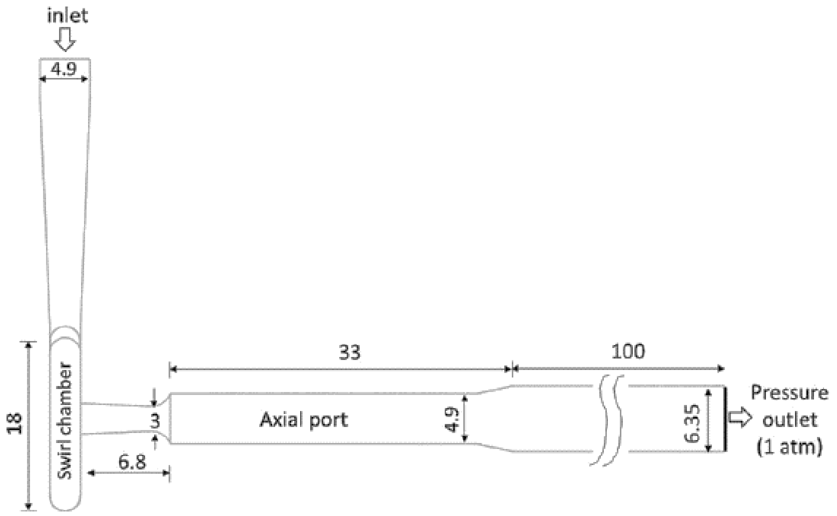

(b)

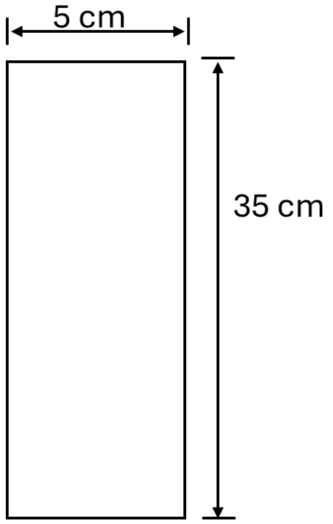

Figure S1: Geometry of (a) Vortex doide (Throat diameter: 3 mm, total volume: 0.7 mL) (b) Bubble column.

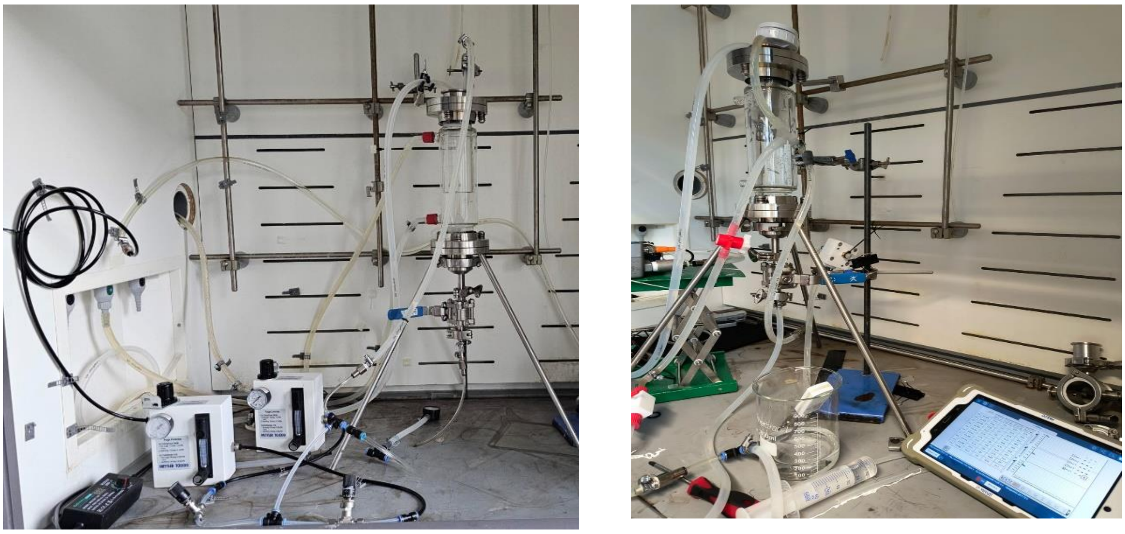

(a)

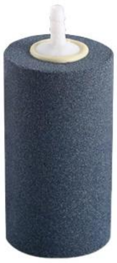

(b)

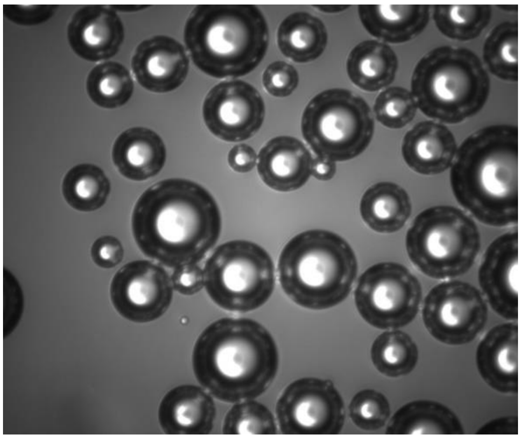

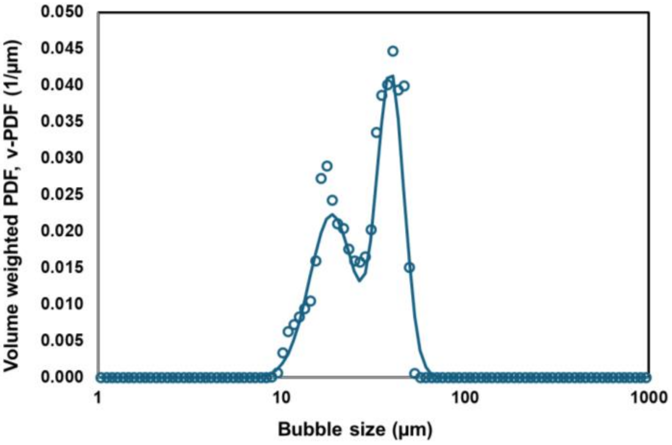

(c) (d)

Figure S2: Photographs of (a) Experimental setup (b) Sparger (c) Image of microbubbles (d) Volume-weigthed probability density function (1/µm) of microbubbles using SOPAT.

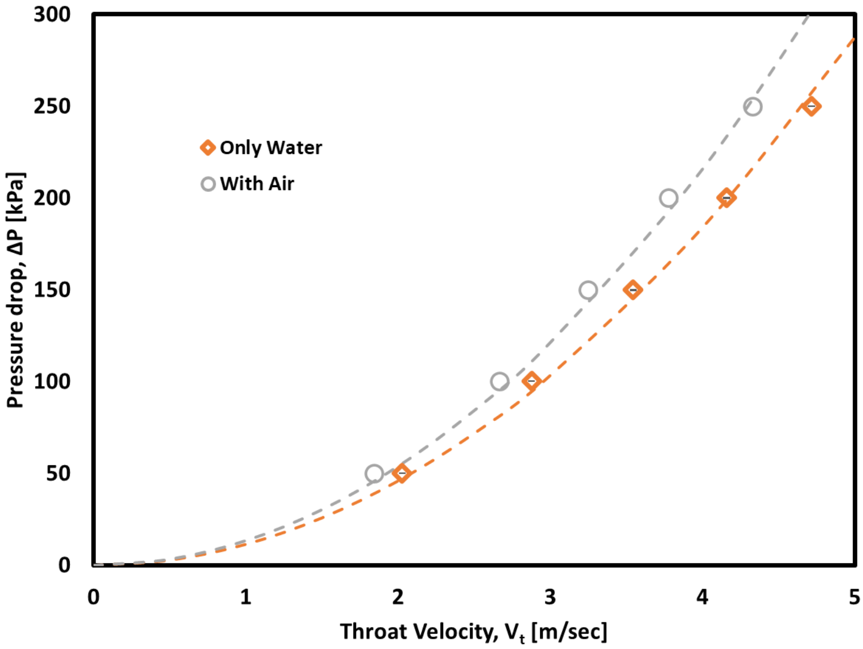

Figure S3: Pressure drop characteristics of VD. Symbols denote experimental data and dashed lines denote correlations [Eu = 23 (only water) and Eu = 27 (with Air)]

Table S1: Initial and Final DO values for degassing experiments

Pressure (kPa)	Initial DO (mg/L)	Final DO (mg/L)
150	8.64	8.46
200	8.76	8.28
250	8.61	8.37

Table S2: Physical properties of system considered in this work

Physical Parameters	Density (kg/m^3)	Viscosity (Pa.s)	DO (mg/L)	Temperature (°C)	Surface tension (N/m)	Conductivity (μS/cm)
Water	998	1×10^{-3}	8.6 ± 0.5	20 ± 2	0.072	0.055
Air (~21% oxygen)	2.35 (at 200 kPa)	1.821×10^{-5}	-	23 ± 2	-	-

Table S3: Operating conditions

Pressure (kPa)	Liquid Flow rate (LPM)	Temp (°C)	Air Flow Rate (LPM)
150	1.5	20 ± 2	0.2
200	1.76	20 ± 2	0.2
250	2	20 ± 2	0.2

Table S4: Pressure drop data (only water)

ΔP (kPa)	Q (m^3/s) × 10^5	Vt (m/s)	Eu
50	1.43	2.02	23
100	2.03	2.87	
150	2.5	3.53	
200	2.95	4.17	
250	3.33	4.71	

Table S5: Pressure drop data (with air)

ΔP (kPa)	Q (m^3/s) × 10^5	Vt (m/s)	Eu
50	1.30	1.84	27
100	1.88	2.66	
150	2.29	3.24	
200	2.67	3.77	
250	3.06	4.32	

Table S6: Cavitation numbers at different operating conditions

ΔP (kPa)	Outlet pressure, P_2 (Pa)	Vapour pressure, P_v (Pa)	Ca (Cavitation number)
50	101325	2353	4
100	101325	2353	2
150	101325	2353	1.3
200	101325	2353	1
250	101325	2353	0.8

<u>Derivation of Equation 3</u>

The internal pressure of a bubble, P_b, using the Young-Laplace equation is given as:
$P_b = P_A + \frac{4\sigma}{d_b}$

Deriving the effective concentration (C_O^*) in terms of C_{OA}^*:

- Using Henry's law with the bubble's internal pressure: $C_O^* = H\, P_b$
- Substituting for P_b, we get:
  $$C_O^* = H(P_A + \frac{4\sigma}{d_b})$$
- For ambient pressure, P_A, saturated DO concentration is, $C_{OA}^* = H\, P_A$.
- Thus, C_O^* in terms of C_{OA}^* can be written as: $C_O^* = C_{OA}^*\,(1 + \frac{4\sigma}{P_A d_b})$
- $\frac{C_O^*}{C_{OA}^*} = 1 + \frac{4\sigma}{P_A d_B}$

Where,

- C_O^*: Saturation concentration of dissolved oxygen in the presence of a microbubble.
- C_{OA}^*: Saturation concentration of oxygen in water under atmospheric conditions.
- σ: Surface tension of the liquid-gas interface (0.072 N/m at 20°C for air-water).
- P_A: Ambient pressure (usually atmospheric pressure, approximately 101.3 kPa).
- P_b: Pressure in a bubble
- d_b: Bubble diameter
